# Expanding the application of tyrosine: engineering microbes for the production of tyrosine and its derivatives

**DOI:** 10.3389/fbioe.2025.1519764

**Published:** 2025-04-24

**Authors:** Jian Shen, Pengfu Liu, Bin Zhang, Bangce Ye, Shunqing Xu, Weike Su, Xiaohe Chu

**Affiliations:** ^1^ Collaborative Innovation Center of Yangtze River Delta Region Green Pharmaceuticals, Zhejiang University of Technology, Hangzhou, Zhejiang, China; ^2^ College of Bioscience and Bioengineering, Jiangxi Agricultural University, Nanchang, Jiangxi, China; ^3^ East China University of Science and Technology, Shanghai, China

**Keywords:** aromatic compounds, tyrosine, biomanufacturing, microbial fermentation, metabolic engineering

## Abstract

Aromatic compounds are widely used in the fields of medicine, chemical industry, and food, with a considerable market size. Tyrosine, an aromatic amino acid, boasts not only a wide range of applications but also serves as a valuable precursor for synthesizing a diverse array of high-value aromatic compounds. Amid growing concerns over environmental and resource challenges, the adoption of green, clean, and sustainable biotechnology for producing aromatic compounds is gaining increasing recognition as a viable alternative to traditional chemical synthesis and plant extraction methods. This article provides an overview of the current status of tyrosine biomanufacturing and explores the methods for generating derivatives, including resveratrol, levodopa, *p*-coumaric acid, caffeic acid, zosteric acid, tyrosol, hydroxytyrosol, tanshinol, naringenin, eriodictyol, and salidroside, using tyrosine as a primary raw material. Furthermore, this review examines the current challenges and outlines future directions for microbial fermentation for the production of tyrosine and its derivatives.

## 1 Introduction

**FIGURE 1:**
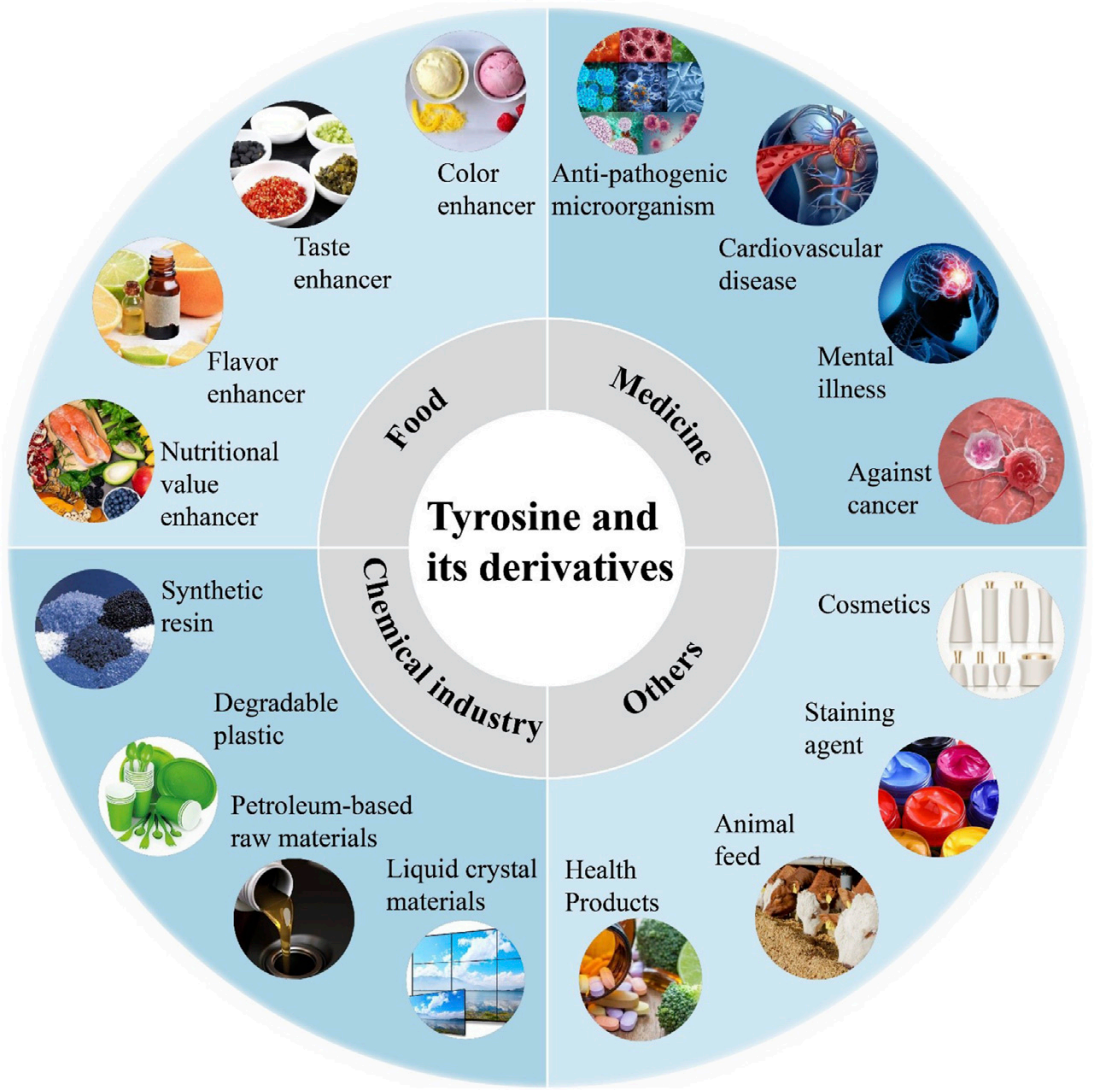
The functions and applications of tyrosine and its derivatives.

Aromatic compounds, which feature a benzene ring, are widely used across industries like chemicals, dyestuffs, medicine, textiles, and food (Effendi and Ng, 2023). This sector is worth billions of dollars (Effendi and Ng, 2023). Aromatic amino acids (AAAs) such as tryptophan, phenylalanine, and tyrosine are crucial for producing various high-value compounds. AAAs are key in applications like food additives, flavor enhancers, pharmaceuticals, and cosmetics (Bongaerts et al., 2001). They can also be transformed into other valuable compounds (Rodriguez et al., 2014), such as violacein from tryptophan, which has antibacterial, antiviral, and antitumor properties (Rodrigues et al., 2013). L-tyrosine, as a high-value amino acid, boasts a wide array of applications. Initially, it was used as a food additive known to enhance brain activity, improve memory, and alleviate restlessness and depression (Deijen and Orlebeke, 1994) (Figure 1). The comprehensive use of tyrosine to treat patients with phenylketonuria offers significant advantages (Rohr et al., 1998). Tyrosine is considered a high-value amino acid in biotechnology due to its ability to be converted into a variety of valuable chemicals, including caffeic acid, *p*-coumaric acid, zosteric acid, levodopa (L-DOPA), and etc.

Various methods are available for preparing tyrosine, including chemical synthesis, enzymatic biocatalysis, and microbial fermentation (Lutke-Eversloh et al., 2007). Chemical synthesis typically involves the acid-base hydrolysis of wheat and corn gluten, yielding tyrosine (Lutke-Eversloh et al., 2007). However, this process is intricate, costly, and environmentally harmful owing to severe pollution. Enzymatic biocatalysis typically uses tyrosine phenol lyase, which requires pyridoxal phosphate, to catalyze a series of α, β-elimination, β-substitution, and racemization reactions, allowing for the synthesis of tyrosine from substrates including phenol, pyruvate, and ammonia. Heterologous expression of tyrosine phenol lyase from *Symbiobacterium toebii* has achieved a tyrosine titer of 131 g/L in a 2.5-L fermentor (Kim et al., 2007). However, enzymatic biocatalysis is challenging for industrial applications because it requires relatively expensive raw materials and complex reaction conditions. Microbial fermentation involves using biomass, such as glucose and cellulose, as substrates and engineered high-efficiency strains as “manufacturing factories” to synthesize tyrosine, thereby changing the high-consumption, high-pollution, and unsustainable production model. With the depletion of fossil resources and increasing emphasis on environmental protection, the use of energy-saving, environmentally friendly, and sustainable biotechnological methods to replace existing chemical synthesis methods for producing aromatic compounds has gradually become a research hotspot (Kim et al., 2023).

## 2 Advances in the microbial biosynthesis of tyrosine

**FIGURE 2:**
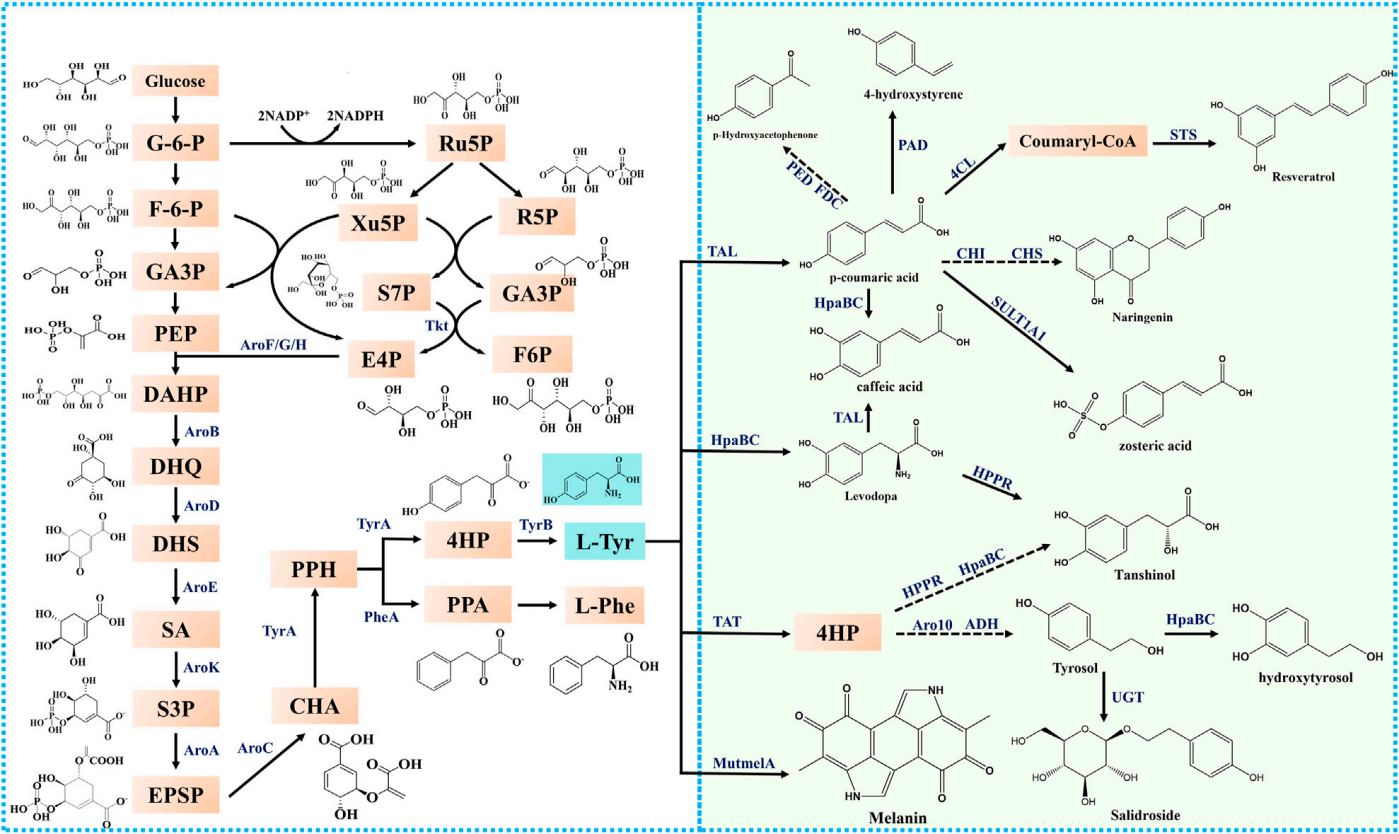
The metabolic pathways of tyrosine and its derivatives in microorganisms. G-6-P, glucose-6-phosphate; F-6-P, fructose-6- phosphate; GA3P, glyceraldehyde-3-phosphate; PEP, phosphoenolpyruvate; Ru5P, ribulose-5-phosphate; Xu5P, xylulose-5-phosphate; R5P, ribose-5-phosphate; S7P, sedoheptulose-7-phosphate; E4P, erythrose-4-phosphate; DAHP, 3-deoxy-D-arabino-heptulosonate-7-phosphate; DHQ, 3-dehydroquinic acid; DHS, 3-dehydroshikimate; SA, shikimic acid; S3P, shikimate-3-phosphate; EPSP, 5-enolpyruvoylshikimate 3-phosphate; CHA, chorismic acid; L-Phe, L-phenylalanine; L-Tyr, L-tyrosine; L-Trp, L-tryptophan 4-HP: 4-Hydroxy-phenylpyruvate; PPA: Phenyl-Pyruvate; PPH: Prephenate; Tkt: transketolase; AroF/G/H, 3-deoxy-D-arabino-heptulosonate-7-phosphate synthase; AroB, 3-dehydroquinate synthase; AroD, 3-dehydroquinate dehydratase; AroE, dehydroshikimate reductase; AroK, shikimate kinase; AroA, 5-enolpyruvylshikimate-3-phosphate synthetase; AroC, chorismate synthase; TyrA, chorismate mutase/prephenate dehydrogenase; PheA, chorismate mutase; TyrB, tyrosine aminotransferase; TAT, tyrosine aminotransferase; TAL, tyrosine ammonia-lyase; HpaB, 4-hydroxyphenylacetate 3-monooxygenase; HpaC, 4-hydroxyphenylacetate 3- monooxygenase, reductase component; 4CL, 4-coumarate:CoA ligase; FDC, encoding ferulic acid decarboxylase; PED: encoding (S)1-phenylethanol dehydrogenase; UGT, UDP-glucosyltransferases; PAD, phenolic acid decarboxylase; MutmelA, tyrosinase; STS, stilbene synthase; CHI, chalcone isomerase; CHS, chalcone synthase; SULT1A1, sulfotransferase; HPPR, Hydroxyphenylpyruvate reductase; Aro10, 2-ketoisovalerate decarboxylase; ADH, alcohol dehydrogenase.

Tyrosine is synthesized via the naturally occurring tyrosine synthesis pathway in microorganisms. In *Escherichia coli*, glucose is first transported across the membrane via the phosphotransferase system and phosphorylated in the cell to form glucose-6-phosphate (G-6-P), which flows into the glycolytic pathway (EMP) and pentose phosphate pathway. The precursor phosphoenolpyruvate (PEP) was generated via the EMP pathway. In the pentose phosphate pathway, another precursor, erythrose-4-phosphate (E4P), is synthesized by transketolase. The generated PEP and E4P were catalyzed by 3-deoxy-D-arabinoheptulose-7-phosphate (DAHP) synthase to form DAHP, which in turn generated 3-dehydroquinic acid (DHQ) via redox, β-elimination, and hydroxyaldehyde condensation by 3-dehydroquinate synthase (encoded by *aroB*). DHQ is then catalyzed by 3-dehydroquinate dehydratase to form 3-dehydroshikimic acid (DHS), which catalyzes the synthesis of shikimic acid (SA) by shikimate dehydrogenase (encoded by *aroE*). SA is catalyzed by shikimate kinase (encoded by *aroK* and *aroL*) to form shikimate-3-phosphate (S3P), which is in turn catalyzed by 5-enolpyruvoylshikimate 3-phosphate (EPSP) synthase to form EPSP. EPSP is catalyzed by chorismic acid (CHA) synthase (encoded by *aroC*) to form CHA, which in turn catalyzes prephenate (PPH). The synthesis of tyrosine is subsequently processed by the formation of 4-hydroxyphenylpyruvate (4-HP) from PPH catalyzed by TyrA, followed by the synthesis of tyrosine from 4-HP catalyzed by tyrosine aminotransferase (encoded by *tyrB*) (Figure 2).

In recent years, a large number of genetic modifications and optimizations of the tyrosine metabolism pathway have been conducted through metabolic engineering and synthetic biology, mainly focusing on blocking competitive pathways, relieving feedback inhibition, increasing the precursor supply, and overall combinatorial regulation (Table 1). The phenylalanine and tryptophan synthesis pathways are the main competitive pathways for tyrosine synthesis. Blocking these two pathways can divert more carbon metabolic flux toward the tyrosine synthesis pathway, thereby increasing tyrosine production. Olson et al. (2007) used an industrial *E. coli* strain producing phenylalanine as the parent strain and transformed it into a strain that produced tyrosine through genetic modification. The phenylalanine biosynthesis pathway was disrupted through the inactivation of *pheA*, which encodes bifunctional chorismate mutase/prephenate dehydratase, and *pheL*, which encodes the leader peptide. Using 3-(N-morpholino) propanesulfonic acid (MOPS) medium for fermentation, and simultaneously supplementing with 10 μg/mL of phenylalanine, the tyrosine titer reached 180 ppm in *E. coli* strain DPD4193. Patnaik et al. (2008) evaluated an engineered *E. coli* strain using fed-batch fermentation. Through fermentation optimization, such as the selection of medium components, optimization of glucose supplementation, and choice of defoaming agents, the *E. coli* cell factory reached a tyrosine concentration of 55 g/L in a 200-L fermentor after 48 h. Furthermore, by manipulating key genes such as *aroG*, *tyrA*, *tktA*, *ppsA*, *aroA*, *aroB*, *aroC*, *aroE*, *aroL*, *tyrR*, *trpE*, *adhE*, *pflB*, and *ldhA*, and engineering the specific transmembrane system through the expression of YddG while disrupting AroP and TyrP, as well as expressing phosphoketolase (encoded by *fpk*) from *Bifidobacterium adolescens*, enhancing cofactor supplementation with NADH, and deleting *pta*-*ack* and *poxB* to block the acetic acid synthesis pathway resulted in strain *E. coli* HGD, which produced 92.5 g/L of L-tyrosine in a 5-L fermenter, with a yield of 0.266 g/g glucose (Ping et al., 2023). This is currently the highest reported concentration of tyrosine obtained through microbial fermentation, which has a high application value and is expected to promote the biosynthesis of tyrosine derivatives.

**TABLE 1:** Microorganisms for tyrosine and its aromatic derivatives production developed by metabolic engineering.

Product	Microorganism	Substrate	Titer	Fermentation mode	References
L-tyrosine	*E. coli*	Glucose	55 g/L	Fed-batch	Patnaik et al. (2008)
L-tyrosine	*C. glutamicum*	Sucrose	26 g/L	Fed-batch	Ikeda and Katsumata (1992)
L-tyrosine	*E. coli*	Glucose	2.2 g/L	Shake-flask	Juminaga et al. (2012)
L-tyrosine	*E. coli*	Glucose	92.8 g/L	Fed-batch	Ping et al. (2023)
4-Hydroxystyrene	*E. coli*	Glucose	0.4 g/L	Fed-batch	Qi et al. (2007)
4-Hydroxystyrene	*E. coli*	Glucose	355 mg/L	Shake-flask	Kang et al. (2015)
*p*-Hydroxystyrene	*P. putida S12*	Glucose	17.6 g/L	Fed-batch	Verhoef et al. (2009)
Zosteric acid	*E. coli*	L-tyrosine	5 g/L	Shake-flask	Jendresen and Nielsen (2019)
*p*-Hydroxyacetophenone	*E. coli*	Glucose	1445.3 mg/L	Fed-batch	Lu et al. (2023)
Naringenin	*S. cerevisiae*	*p*-coumaric acid	2.05 g/L	Fed-batch	Mao et al. (2023)
Eriodictyol	*E. coli*	L-tyrosine	107 mg/L	Shake-flask	Zhu et al. (2014)
Eriodictyol	*C. glutamicum*	tyrosine	14.10 mg/L	Fed-batch	Wu et al. (2022)
Salidroside	*E. coli*	Glucose	56.9 mg/L	Fed-batch	Bai et al. (2014)
Salidroside	*R. crenulata*	Leaf	26.48 mg/g	Cell suspension cultures	Shi et al. (2013)
4-Hydroxymandelic acid	*E. coli*	Glucose and Xylose	15.8 g/L	Fed-batch	Li et al. (2016)
Melanin	*E. coli*	Glucose	3.22 g/L	Fed-batch	Chávez-Béjar et al. (2013)
Melanin	*Empa 655*	L-tyrosine	27.98 g/L	Shake-flask	Ribera et al. (2019)
Salvianic acid A	*E. coli*	Glucose	5.6 g/L	Fed-batch	Zhou et al. (2017)
Salvianic acid A	*E. coli*	Phenylpyruvate	8 g/L	Whole-cell catalysis	Li et al. (2019)
Tyrosol	*E. coli*	L-Tyrosine	8.71 mM	Whole-cell catalysis	Xue et al. (2017)
Tyrosol	*E. coli*	Glucose	926.9 mg/L	Shake-flask	Bai et al. (2014)
Tyrosol	*E. coli*	L-Tyrosine	0.5 mM	Shake-flask	Satoh et al. (2012a)
Hydroxytyrosol	*E. coli*	L-Tyrosine	1243 mg/L	Whole-cell catalysis	Li et al. (2018)
Hydroxytyrosol	*E. coli*	Glucose	0.08 mM	Shake-flask	Satoh et al. (2012b)
Caffeic acid	*E. coli*	*p*-Coumaric acid	3.82 g/L	Whole-cell catalysis	Huang et al. (2013)
Caffeic acid	*E. coli*	Glucose	766.68 mg/L	Shake-flask	Huang et al. (2013)
Caffeic acid	*E. coli*	Glucose	106 mg/L	batch	Zhang and Stephanopoulos (2013)
*p*-Coumaric acid	*E. coli*	Glucose	1.93 g/L	96-Deep well plate	Rodriguez et al. (2015)
*p*-Coumaric acid	*E. coli*	Sugar mixtures	355.87 μM	Shake-flask	Vargas-Tah et al. (2015)
*p*-Coumaric acid	*P. putida*	Glucose	1.7 g/L	Fed-batch	Nijkamp et al. (2007)
L-DOPA	*E. coli*	Catechole, Pyruvate	29.8 g/L	Whole-cell catalysis	Lee et al. (1996)
L-DOPA	*E. coli*	Glucose	8.67 g/L	Fed-batch	Wei et al. (2016)
L-DOPA	*E. coli*	L-tyrosine	9.47 g/L	Fed-batch	Lee and Xun (1998)

## 3 Advances in the biosynthesis of tyrosine derivatives

Natural biosynthesis and derivative pathways of AAAs in organisms provide alternative bio-based routes to traditional chemical synthesis methods for aromatic compound production (Fengli et al., 2017). By leveraging heterologous metabolic pathway assembly techniques, tyrosine can serve as a precursor for the synthesis of a wide variety of chemicals, including L-DOPA, *p*-coumaric acid, caffeic acid, tyrosol, hydroxytyrosol, melanin, and naringenin (Table 1). The synthesis of these compounds using tyrosine as a substrate may require one reaction step, two reaction steps, or even three or more metabolic reactions. Below, we will review the research progress on the synthesis of these tyrosine derivatives based on the number of metabolic reactions required.

### 3.1 One-step tyrosine-derived products

#### 3.1.1 L-DOPA

L-DOPA, the most effective drug for treating Parkinson’s disease, has good tolerance, improving bradykinesia, relieving the main symptoms, and prolonging the lifespan of patients (Min et al., 2015). As the global population ages, the demand for L-DOPA has surged, increasing the need for more efficient production methods. Currently, microbial synthesis of L-DOPA offers considerable advantages and has been widely adopted.

The pioneering work in microbial L-DOPA production began with Lee et al. (1996), who cloned a thermostable tyrosine phenol lyase from *Symbiobacterium* and overexpressed it in *E. coli*. Using catechol and pyruvate as substrates, they achieved a L-DOPA titer of 29.8 g/L after 6 h under optimal catalytic conditions. Building on this, Jang et al. (Lee and Xun, 1998) engineered *E. coli* strain W to constitutively express 4-hydroxyphenylacetic acid 3-hydroxylase (encoded by *hpaBC*), which produced 9.47 g/L of L-DOPA using tyrosine as the substrate. Further advancements were made by Muñoz et al. (2011), who synthesized L-DOPA in *E. coli* VH33tyrR_DOPA by overexpressing the *hpaBC* genes. After 50 h of fed-batch fermentation using glucose as the substrate, the L-DOPA titer reached 1.51 g/L. Wei et al. (2016) expanded on this approach by expressing the *hpaBC* genes on a plasmid in *E. coli* BW25113. They knocked out key transcriptional regulators (encoded by *tyrR* and *csrA*) and altered the glucose transport system by knocking out *ptsHI* and using the P37 promoter to express *galP* and *glk*, which enhanced the flow of PEP. Subsequent modifications to the tyrosine biosynthesis pathway resulted in a titer of 307.4 mg/L in shake-flask fermentation. In an effort to further improve yields, Wei et al. (Wang et al., 2009; Wang et al., 2012) applied Multiplex Automated Genome Engineering (MAGE) technology to screen for mutant strains that produced higher levels of L-DOPA. After 30 rounds of MAGE screening, they identified strains with a significantly improved titer of 614.3 mg/L in shake-flask fermentation. In a 5-L fed-batch fermentation, L-DOPA concentration reached 8.67 g/L after 60 h, using glucose as the substrate. More recently, Eric et al. (Fordjour et al., 2019) engineered *E. coli* BL21 (DE3) by manipulating genes related to tyrosine biosynthesis pathway, deleting the *ptsG*, *crr*, and *pykF* genes, along with the overexpression of *galP* and *glk*, as well as the directed evolution of HpaB, resulting in the creation of strain LP-8. This strain demonstrated the remarkable achievement of producing 25.53 g/L L-DOPA in a 5-L bioreactor, marking the highest reported yield of L-DOPA via microbial fermentation using glucose as the substrate. With ongoing advancements in microbial tyrosine production technology, fermentation-based synthesis of L-DOPA holds significant promise for industrial-scale applications.

#### 3.1.2 *p*-Coumaric acid

*p-*Coumaric acid, a naturally occurring compound in plants, serves as a key precursor for various phenylpropanoids, including lignin, flavonoids, and coumarins. It is a vital biological monomer with diverse biological activities, particularly known for its antioxidant and anticancer properties (Fengli et al., 2017; Nijkamp et al., 2007). These attributes have made it a crucial component in both biological and medical applications (Fengli et al., 2017; Nijkamp et al., 2007).

The biosynthesis of *p*-coumaric acid from tyrosine has been successfully achieved in microbial systems, notably using *E. coli*, *Saccharomyces cerevisiae*, *Corynebacterium glutamicum*, and *Pseudomonas putida* as chassis cells. In *E. coli*, Trotman et al. (Trotman et al., 2007) expressed tyrosine ammonia-lyase (TAL) from *Rhodotorula glutinis* in *E. coli* and employed calcium alginate to immobilize the cells, achieving the conversion of 50 g/L tyrosine into 39 g/L *p*-coumaric acid in a 1-L bioreactor. Similarly, Vargas et al. (Vargas-Tah et al., 2015) enhanced *p*-coumaric acid yield to 355.87 μmol/L in *E. coli* by overexpressing phenylalanine ammonia lyase (PAL) and modifying metabolic pathways to relieve feedback inhibition and reduce by-product formation. Further improvements were achieved by Qiu et al. (2024) achieved significant improvements by engineering a more soluble variant of AtC4H and enhancing the cofactor supply in *E. coli*, resulting in a strain capable of producing 3.09 g/L of *p*-coumaric acid.

Beyond *E. coli*, other microbial platforms have been engineered for *p*-coumaric acid production. In *S. cerevisiae*, Rodriguez et al. (2015) successfully synthesized *p*-coumaric acid by knocking out genes involved in aromatic alcohol synthesis and overexpressing TAL. *C. glutamicum* was engineered to produce *p*-coumaric acid from tyrosine, with further enhancements achieved through manipulating genes related to tyrosine biosynthesis pathway using site-direct mutation (AroF^S188C^, TrpE^P304S^) and start codon replacement techniques (*pheA* ^A1G^ and *aroK* ^G1A^) (Mutz et al., 2023). Through additional inactivation of the phenylpropanoid degradation pathway, reduction of anthranilate synthase activity, the final strain produced *p*-coumaric acid at a titer of 661 mg/L (Mutz et al., 2023). Similarly, *P. putida* has been engineered for *p*-coumaric acid production. Nijkamp et al. (2007) expressed the *pal* encoding PAL, enabling *p*-coumaric acid synthesis. By inactivating *fcs*, the gene encoding the first enzyme in the *p*-coumaric acid catabolic pathway, they reduced its degradation. Further metabolic optimization, including constructing a phenylalanine auxotrophic strain to minimize cinnamic acid by-production and optimizing fermentation conditions, led to a *p*-coumaric acid titer of 1.7 g/L, with a molar ratio of *p*-coumaric acid to cinnamic acid of 85:1. These advancements highlight the successful implementation of plant-derived *p*-coumaric acid biosynthesis pathways into microbial chassis cells. Microbial fermentation provides an efficient, sustainable, and environmentally friendly approach for *p*-coumaric acid production, offering promising applications in biotechnology and industry.

#### 3.1.3 Melanin

Melanin serves various functions such as ultraviolet absorbents, amorphous semiconductors, cation exchangers, and absorbers of X- and Y-rays (Chávez-Béjar et al., 2013). It plays a crucial protective role by shielding the skin from UV radiation and oxidative stress (Del Bino and Bernerd, 2013; Del Bino et al., 2018; Fajuyigbe and Young, 2016), acting as the primary physiological defense against solar radiation (D'Orazio et al., 2006; Scott et al., 2012). The biosynthesis of melanin involves several key components: tyrosine, the primary precursor; tyrosinase, the rate-limiting enzyme that catalyzes the conversion of tyrosine into melanin; and oxygen, which participates in the oxidation process. The synthesis pathway follows these steps: 1. Tyrosine is oxidized to dopaquinone by tyrosinase. 2. Dopaquinone then undergoes self-oxidation to produce dopamine and dopachrome. Subsequently, dopamine serves as a substrate for tyrosinase, leading to the production of dopachrome. 3. The reaction products of dopachrome, namely 5,6-dihydroxyindole and 5,6-dihydroxyindole-2-carboxylic acid, undergo a series of oxidation reactions, ultimately resulting in the formation of eumelanin, the primary component of skin pigmentation. 4. In the presence of cysteine or glutathione, dopaquinone is transformed into cysteinyldopa, ultimately leading to the production of brown-black melanin.

To enhance melanin biosynthesis, metabolic pathways have been integrated into prokaryotic chassis cells, enabling heterologous *de novo* production. Chávez-Béjar et al. (2013) expressed the *mel* gene encoding tyrosinase from *Rhizobium etli* in *E. coli* and optimized the tyrosine biosynthetic pathway using glucose as the sole carbon source. After 120 h of fermentation, the strain produced melanin at a titer of 3.2 g/L. Similarly, Manivasagan et al. (2013) extracted and characterized melanin from the marine actinomycete *Actinoalloteichus* sp. MA-32. By optimizing glycerol, L-tyrosine, NaCl, and trace salt concentrations through a central composite design, they achieved a final melanin yield of 0.1 g/L. Kiran et al. (2017) screened multiple strains for melanin production and ultimately selected *Pseudoalteromonas* strain WH001 55, which, after 6 days of fermentation, yielded 7.6 g/L melanin. Beyond bacteria, fungi have demonstrated superior melanin biosynthetic potential. Ribera et al. (2019) evaluated different fungal strains using L-tyrosine as a precursor and identified a honey fungus strain (*Empa 655*) that achieved an impressive melanin titer of 27.98 g/L.

A comparative analysis of these studies underscores the efficiency of fungal strains for melanin synthesis. Current research has primarily focused on isolating and screening naturally occurring strains. However, unlocking the regulatory mechanisms governing melanin biosynthesis could further enhance production efficiency. By elucidating these pathways, researchers may leverage gene editing technologies to optimize strain performance and maximize melanin yield. This approach holds great promise for developing more efficient and scalable melanin production systems.

### 3.2 Two-step tyrosine-derived products

#### 3.2.1 Caffeic acid

Caffeic acid, widely found in plants, serves as a precursor for high-performance thermoplastics, offering high mechanical strength and low softening temperatures. In addition to its industrial applications, it exhibits various pharmacological activities, including antioxidative, anti-inflammatory, antiviral, antidepressant, and antidiabetic effects. Its derivative, caffeic acid phenethyl ester, has shown promise in alleviating liver cirrhosis and exhibiting antitumor properties, particularly against prostate cancer (Kassa et al., 2021; Khan et al., 2021; Sabahi et al., 2023).

As a high-value aromatic compound, caffeic acid can be synthesized by the hydroxylation of coumaric acid or the deamination of L-DOPA. To achieve efficient microbial production, various metabolic engineering strategies have been employed. Zhang and Stephanopoulos (2013) engineered a high-yield tyrosine-producing strain of *E. coli* by expressing *TAL* gene from *R. glutinis* and the 4-coumarate-3-hydroxylase gene from *Saccharothrix espanaensis*. Both genes were codon-optimized to construct a strain for caffeic acid production. The optimal gene dosage was determined by optimizing the plasmid copy number, and the yield of caffeic acid was enhanced using different media, including MOPS, synthetic, and rich media. Following fermentation for 4 days in a 2-L fermenter with glucose as the substrate and synthetic medium, the concentration of caffeic acid reached 106 mg/L. In a different approach, Huang et al. (2013) overexpressed *hpaBC*, encoding 4-hydroxyphenylpropionate-3-hydroxylase, in *E. coli* and utilized whole cells to catalyze the conversion of *p*-coumaric acid into caffeic acid. This method resulted in a significantly higher yield of 3.82 g/L. Kawaguchi et al. (2017) further improved microbial caffeic acid production by introducing *hpaBC* from *Streptomyces* sp. WK-5344 and *fevV* from *Pseudomonas aeruginosa* into *E. coli*. They discovered that limiting glucose concentration reduced by-product formation and fermentation inhibitors, ultimately increasing the caffeic acid titer to 233 mg/L through optimized fermentation conditions and simultaneous saccharification. A more recent study by Sakae et al. (2023) achieved the production of 6.17 g/L of caffeic acid by overexpressing RgTAL from *R. glutinis*, SeC3H from *S. espanaensis*, HpaBC from *P. aeruginosa*, and an endogenous anti-feedback chorismate mutase/prephenate dehydrogenase. Notably, Wang et al. (2023) reported the highest caffeic acid production to date, reaching an impressive 7.92 g/L in *E. coli*. This breakthrough was achieved through a combination of strategies, including the deletion of competing metabolic pathways, optimization of cofactor FAD supplementation, and overexpression of a newly identified transporter, YcjP.

These advancements highlight the effectiveness of metabolic engineering in enhancing caffeic acid biosynthesis. With continued improvements in pathway optimization, cofactor regulation, and transporter engineering, microbial production of caffeic acid holds great promise for industrial and pharmaceutical applications.

#### 3.2.2 Zosteric acid

Zosteric acid, a sulfated phenolic acid derived from eelgrass, exhibits strong antifouling properties against various marine organisms. This makes it a promising eco-friendly alternative to synthetic antifouling agents commonly used in marine coatings (Alfieri et al., 2024; Sævdal Dybsland et al., 2021).

The biosynthesis of zosteric acid can originate from both tyrosine and *p*-coumaric acid, which serve as key precursor molecules in its metabolic pathway. Tyrosine is first converted into *p*-coumaric acid via TAL. Subsequently, *p*-coumaric acid undergoes sulfation in the presence of 3′-phosphoadenosine 5′-phosphosulfate (PAPS), a universal sulfate donor, under the catalysis of PAPS-dependent aryl sulfotransferases, leading to the formation of zosteric acid (Olsen et al., 2016). These metabolic pathways have been successfully introduced into model microorganisms, enabling the heterologous synthesis of zosteric acid.

Several studies have demonstrated microbial production of zosteric acid through metabolic engineering approaches. Jendresen and Nielsen (2019) demonstrated *de novo* biosynthesis of zosteric acid by overexpressing *SULT1A1* from *Rattus norvegicus* and TAL from *Flavobacterium johnsoniae* in both *E. coli* and *S. cerevisiae*. Additionally, by optimizing the sulfur metabolic pathway to enhance PAPS availability, the engineered strain achieved a zosteric acid yield of 5.0 g/L when supplemented with tyrosine. Zhang et al. (2023) further advanced microbial production by developing a recombinant *E. coli* strain capable of synthesizing zosteric acid using glucose and glycerol as substrates. Through genetic modifications—such as increasing PAPS supply and overexpressing *TAL*, adenosine 5′-phosphosulfate kinase, and ATP sulfurylase—the optimized strain produced 1.52 g/L of zosteric acid alongside 1.30 g/L of *p*-hydroxycinnamic acid. These findings highlight the potential of genetically engineered *E. coli* strains for the biosynthesis of low-yield antifouling agents and suggest a viable approach for improving production efficiency.

Despite these advances, microbial production of zosteric acid remains in its early stages. A key challenge lies in the inherent preservative properties of zosteric acid, which can negatively affect microbial growth and limit overall production efficiency. Future research should focus on optimizing metabolic engineering strategies, enhancing microbial tolerance to zosteric acid, and improving pathway regulation to achieve higher yields in microbial systems. Overcoming these challenges will be crucial for the sustainable and scalable production of zosteric acid as a bio-based antifouling agent.

#### 3.2.3 4-Hydroxystyrene

4-Hydroxystyrene holds significant importance as a commodity chemical, finding applications in the manufacture of various industrially significant petroleum-derived raw materials, as well as flavorings for polymers, resins, elastomers, and adhesives (Gargatte et al., 2021). Its ability to form linear polymers and excellent solubility in organic solvents make it a desirable reagent for synthesizing coatings for electronic devices, such as photoresists (Gargatte et al., 2021).

The microbial production of 4-hydroxystyrene integrates multiple metabolic pathways, with the most cost-effective route relying on glucose as a carbon and energy source. Qi et al. (2007) successfully established a biosynthetic pathway for 4-hydroxystyrene in *E*. *coli*, achieving a production titer of 0.4 g/L in fed-batch bioreactors. Kang et al. (2015) proposed a pathway utilizing *L*-tyrosine as a precursor, converting it to 4-hydroxystyrene through a series of enzyme-catalyzed steps. Their engineered *E. coli* strain produced 355 mg/L of 4-hydroxystyrene from 15 g/L glucose in shake-flask cultivation. Jung et al. (2013) further optimized this pathway, achieving an 88.7% conversion efficiency from *p*-coumaric acid to 4-hydroxystyrene. Meanwhile, Verhoef et al. (2009) introduced the biosynthetic pathway into *P*. *putida* and developed a two-phase fermentation process, leveraging the strain’s high tolerance to toxic organic solvents. This approach resulted in a significantly improved 4-hydroxystyrene production of 2.5 g/L from glucose. These advancements highlight the potential of metabolic engineering in enhancing the microbial production of both 4-hydroxystyrene. By refining key biosynthetic pathways and optimizing host strains, researchers can further improve production yields, paving the way for more sustainable and scalable biotechnological processes.

### 3.3 Multi-step tyrosine-derived products

#### 3.3.1 Tyrosol

Tyrosol, a natural phenolic compound found in *Rhodiola rosea*, plays a crucial role in pharmaceutical and organic synthesis. It serves as an intermediate in the production of β1 receptor blockers such as metoprolol and betaxolol, which are widely used to treat hypertension and glaucoma (Satoh et al., 2012a; Xue et al., 2017). Additionally, tyrosol is a precursor for hydroxytyrosol, a potent antioxidant with various health benefits, including cardiovascular disease prevention, osteoporosis mitigation, and anti-inflammatory properties. Beyond its pharmacological applications, tyrosol also enhances the flavor of Japanese rice wine (Guzowski et al., 2024; Liu et al., 2007).

The biosynthesis of tyrosol follows a multistep catalytic pathway using tyrosine as a precursor. Satoh et al. (2012a) established a microbial biosynthetic route for tyrosol in *E*. *coli* by introducing the tyrosine decarboxylase gene from *Papaver somniferum* and the tyramine oxidase gene from *Micrococcus luteus*. To improve efficiency, they deleted *feaB*, encoding phenylacetaldehyde dehydrogenase, to reduce the formation of the by-product *p*-hydroxyphenylacetic acid. This modification increased tyrosol production to 1.22 mM, achieving a yield of 0.5 mM in M9Y medium after two days of fermentation with glucose as the substrate. Further improvements in microbial production were achieved by Bai et al. (2014), who constructed an alternative biosynthetic pathway for tyrosol by overexpressing phenylpyruvate decarboxylase from *S*. *cerevisiae* and inactivating *feaB*. These modifications resulted in a tyrosol yield of 128.3 mg/L, which was further increased to 926.9 mg/L by enhancing genes involved in the tyrosine biosynthesis pathway. Xue et al. (2017) further optimized tyrosol production in *E. coli* by integrating multiple strategies, including overexpression of the *ARO10* gene, knockout of *feaB*, and augmentation of aromatic amino acid aminotransferase expression from *S. cerevisiae*. By optimizing culture conditions using glucose as a substrate, they achieved a tyrosol yield of 4.15 mM in shake-flask fermentation over 48 h. Additionally, by employing whole-cell catalysis with tyrosine as the substrate and supplementing the medium with an additional 10 mM tyrosine, they obtained an impressive tyrosol yield of 8.71 mM after 20 h, demonstrating a high conversion rate of 87.1%.

These studies highlight the potential of metabolic engineering for efficient microbial production of tyrosol. However, challenges such as pathway bottlenecks, by-product formation, and substrate availability remain key obstacles to further enhancing yield. Future research should focus on optimizing metabolic flux, engineering cofactor balance, and improving strain tolerance to increase the industrial feasibility of microbial tyrosol production.

#### 3.3.2 Hydroxytyrosol

Hydroxytyrosol, a powerful antioxidant primarily found in olives, exhibits superior free radical scavenging efficacy compared to vitamins E and C (Terracina et al., 2022). Its potential health benefits include the prevention of cardiovascular diseases, metabolic disorders, cancer, inflammation, and bacterial infections. With excellent bioavailability and minimal toxicity, hydroxytyrosol is considered a promising therapeutic compound for applications in the food and pharmaceutical industries (Achmon and Fishman, 2015; Aunon-Calles et al., 2013).

Microbial synthesis offers a sustainable approach for hydroxytyrosol production. Satoh et al. (2012b) pioneered the development of an *E*. *coli* strain capable of producing hydroxytyrosol by first disrupting *feaB* to reduce by-product formation. They then introduced a synthetic pathway from L-DOPA to hydroxytyrosol by expressing codon-optimized L-DOPA decarboxylase and tyrosinase from *Streptomyces griseus*. To further enhance hydroxytyrosol production, they engineered an alternative biosynthetic route from tyrosine or glucose by incorporating tyrosine hydroxylase, pterin-4a-carbinolamine dehydratase, and dihydropteridine reductase. Using glucose as a substrate, this engineered strain achieved a hydroxytyrosol yield of 0.08 mM after 3 days of shake-flask fermentation. Building on these efforts, Li et al. (2018) developed a high-yield microbial synthesis platform for hydroxytyrosol by first optimizing tyrosol production. They constructed a tyrosol biosynthetic pathway in *E. coli* by overexpressing phenylpyruvate decarboxylase and alcohol dehydrogenase from *S*. *cerevisiae*, significantly increasing tyrosol yield. Based on this engineered strain, they further introduced *hpaBC* genes to convert tyrosol into hydroxytyrosol. To improve production efficiency, they employed several optimization strategies: enhancing the transamination of tyrosine to 4-HP by removing NH_4_Cl from the culture medium, preventing hydroxytyrosol oxidation by supplementing ascorbic acid, and implementing biphasic fermentation with suitable extraction agents to reduce hydroxytyrosol toxicity to the cells. These optimizations led to a significant increase in hydroxytyrosol yield, reaching 1243 mg/L with tyrosine as the substrate and 647 mg/L with glucose as the substrate. These studies effectively showcase the feasibility of employing prokaryotes to synthesize natural alcohols and highlight their broad potential applications.

#### 3.3.3 Tanshinol

Tanshinol, a water-soluble component of tanshinone with a catechol structure, is a key bioactive compound in traditional Chinese medicine, widely used for preventing and treating vascular diseases (Zhou et al., 2017). It shows potential in inhibiting arterial thrombosis, alleviating liver and myocardial injuries, restoring brain nerve damage, and possesses anti-inflammatory, antitumor, antioxidant, and anti-fibrosis properties, while also serving as a precursor for synthesizing pharmacologically active compounds (Xue et al., 2022; Subedi and Gaire, 2021; Sun et al., 2022).

Microbial synthesis provides a promising alternative for tanshinol production. Yao et al. (2013) successfully engineered an *E. coli* strain by expressing the *hpaBC* and *ldh* genes from *Lactobacillus pentosus*, optimizing the metabolic pathway and constructing an engineered strain with an impressive tanshinol titer of 7.1 g/L. Similarly, Zhou et al. (2017) modularized and assembled the pertinent genes responsible for the tanshinol synthesis pathway onto the chromosome of *E. coli*, achieving a tanshinol titer of 5.6 g/L. In addition to microbial biosynthesis, hybrid chemical-enzymatic approaches have also been explored. Rao and Roth (2006) employed a combination of chemical and enzymatic approaches to synthesize tanshinol. They first treated L-DOPA with trifluoroacetic anhydride using the Steglich reaction, yielding an unstable trifluorolactone intermediate that hydrolyzed into 3,4-dihydroxyphenylacetic acid. This compound was subsequently converted into tanshinol via L-hydroxy acid dehydrogenase. Similarly, Findrik et al. (2005) utilized snake venom oxidase to convert L-DOPA into 3,4-dihydroxyphenylacetic acid, followed by lactate dehydrogenase-mediated transformation into tanshinol. More recently, Wang et al. (Li et al., 2019) developed an efficient whole-cell catalysis system for tanshinol production. Using 4-HP as a substrate, they sequentially introduced D-amino acid dehydrogenase, phenylalanine-4-hydroxylase, and hydroxyphenylacetic acid-3-hydroxylase, ultimately achieving an impressive tanshinol titer of 8 g/L. These studies highlight the feasibility of microbial and enzymatic tanshinol synthesis, demonstrating significant progress in pathway optimization and yield improvement.

#### 3.3.4 Resveratrol

Resveratrol, a natural polyphenolic compound found in grapes, berries, and peanuts, has garnered attention for its health benefits, including cardioprotective effects, anti-inflammatory properties, and cholesterol-lowering abilities (Abo-Kadoum et al., 2022; Cao et al., 2022; Meng et al., 2021; Płachta et al., 2024). Additionally, resveratrol has shown promise in neuroprotection, preventing neurodegenerative diseases, inhibiting cancer cell growth, and inducing apoptosis. Its potential role in longevity and metabolic health is also under investigation, as studies suggest it may extend lifespan in animal models (Lephart, 2021; Ren et al., 2021; Yan et al., 2020).

With increasing demand for resveratrol, microbial fermentation has emerged as a promising alternative for large-scale production. Various microbial hosts, including *E. coli*, *Yarrowia lipolytica*, and *S. cerevisiae* have been extensively studied for their potential in resveratrol production. When *E. coli*, *S. cerevisiae*, and *C. glutamicum* were used as host cells, the yield of resveratrol fermentation with glucose as the substrate remained at the milligram level (Shrestha et al., 2019). However, employing whole-cell catalysis with *p*-coumaric acid and cerulenin as precursors significantly improved resveratrol yield in *E. coli*, reaching 2.3 g/L. Among these microbial hosts, *Y. lipolytica* has demonstrated strong potential for resveratrol production due to its abundant supply of acyl-coenzymes, which serve as precursors for resveratrol biosynthesis. In our previous study, we successfully established a resveratrol synthesis pathway in *Y. lipolytica* (Shang et al., 2023). By enhancing shikimate pathway flux, redirecting carbon metabolism, and increasing the copy number of key biosynthetic genes, we achieved a resveratrol yield of 487.77 mg/L. Furthermore, Liu et al. (2022) optimized *Y. lipolytica* by co-expressing *FjTAL*, *Pc4CL1*, and *VvSTS*, while simultaneously modulating the shikimate pathway to enhance *p*-coumaric acid supply and divert glycolytic flux toward E4P. These metabolic engineering strategies enabled an unprecedented resveratrol yield of 22.5 g/L, marking the highest reported *de novo* production of resveratrol to date.

#### 3.3.5 Naringenin

Naringenin serves as a pivotal precursor in the biosynthesis of numerous valuable flavonoids and is renowned for its diverse nutritional and pharmacological benefits, including antioxidative, anticancer, and anti-inflammatory properties (Wu et al., 2013). Given its broad applications, efficient biosynthetic strategies for naringenin production have garnered significant attention. The biosynthesis of naringenin involves two main pathways: (1) the conversion of L-tyrosine into *p*-coumaric acid via TAL, and (2) the transformation of L-phenylalanine into *p*-coumaric acid through phenylalanine ammonia-lyase (PAL) and cinnamic acid hydroxylase. Once *p*-coumaric acid is formed, it undergoes further enzymatic modifications, ultimately leading to the synthesis of naringenin.

Recent studies have focused on optimizing naringenin biosynthesis to overcome pathway bottlenecks and enhance production yields. Mao et al. (2023) successfully addressed downstream metabolic limitations of *p*-coumaric acid, significantly improving naringenin production. Through fed-batch fermentation, their engineered strain achieved a final naringenin titer of 2.05 g/L. In our previous study, we introduced the naringenin biosynthesis pathway into *Yarrowia lipolytica* and developed a xylose-inducible system to enhance production efficiency (Wei et al., 2020). This approach resulted in a naringenin titer of 715.3 ± 12.8 mg/L during shake-flask cultivation, demonstrating the feasibility of employing *Y. lipolytica* as a microbial chassis for flavonoid production (Wei et al., 2020). These advancements highlight the potential of metabolic engineering and synthetic biology in optimizing naringenin biosynthesis. Future research should focus on improving precursor availability, enhancing enzyme efficiency, and further optimizing fermentation conditions to maximize industrial-scale production.

#### 3.3.6 *p*-Hydroxyacetophenone (*p*-HAP)

*p*-HAP, also known as hydroquinone, is a crucial organic compound widely utilized in pharmaceuticals, fragrances, and various industrial applications. It serves as a precursor for essential compounds such as atenolol and *N*-acetyl-*p*-aminophenol. Beyond its industrial significance, *p*-HAP has demonstrated potential antiviral activity against the hepatitis B virus, while its derivatives exhibit diverse pharmacological effects, including bile secretion stimulation, lipid-lowering properties, anti-inflammatory and anticancer activities, antimicrobial effects, and antipsychotic potential (Zhao et al., 2015).

Despite its broad applications, the natural biosynthetic pathways of *p*-HAP remain unidentified, posing a major challenge for its microbial biosynthesis. Currently, industrial *p*-HAP production primarily relies on chemical synthesis, using phenol as a raw material through acylation and Fries rearrangement. However, these chemical methods involve harsh reaction conditions and high purification costs, limiting their large-scale application. Additionally, extracting *p*-HAP from natural plant sources is impractical due to low yields (Bohlmann and Stöhr, 1980). Therefore, establishing an efficient biosynthetic pathway for *p*-HAP is crucial for sustainable and cost-effective production.

Recent advances in metabolic engineering have opened new avenues for microbial *p*-HAP synthesis. Lu et al. (2023) designed a biosynthetic pathway using tyrosine, an intermediate in the *E. coli* shikimate pathway, as the starting substrate. By systematically enhancing precursor supply and optimizing the host strain, they successfully constructed a microbial production system for *p*-HAP and vanillin in *E. coli*. Their engineered strain achieved a *p*-HAP titer of 1445.3 mg/L under fed-batch fermentation conditions, demonstrating the feasibility of microbial production. These findings highlight the potential of synthetic biology and metabolic engineering in developing sustainable biosynthetic routes for *p*-HAP.

#### 3.3.7 Eriodictyol and salidroside

Eriodictyol is a flavonoid found in plants like lemon verbena, yerba santa, and citrus fruits, known for its antioxidant properties (Guo et al., 2023). It exhibits various health benefits, including anti-inflammatory, anticancer, and neuroprotective effects. Similarly, salidroside, a major bioactive compound in *Rhodiola rosea*, is renowned for its adaptogenic properties, aiding in stress resistance while enhancing cognitive function, alleviating fatigue, and promoting overall wellbeing. Additionally, salidroside possesses antioxidant, anti-inflammatory, and neuroprotective activities (Calabrese et al., 2023; Jin et al., 2022; Liu et al., 2023).

Given their valuable pharmacological properties, the biotechnological synthesis of eriodictyol and salidroside has been actively explored, with tyrosine serving as a key precursor. Zhu et al. (2014) successfully engineered an *E. coli* strain for eriodictyol biosynthesis by coexpressing TAL, 4-coumaroyl-CoA ligase, chalcone synthase, and chalcone isomerase, achieving a titer of 107 mg/L. By carefully balancing metabolic flux and gene expression, they enhanced the efficiency of flavonoid biosynthesis. Meanwhile, Li et al. (2016) focused on optimizing tyrosine metabolism in *E. coli* for the production of 4-hydroxymandelic acid, an important intermediate for synthesizing bioactive phenolic compounds. By expressing the 4-HP dioxygenase gene in a high-yield tyrosine-producing strain, fine-tuning expression levels, and knocking out competing pathway genes (*aspC* and *tyrB*) to minimize by-product formation, they achieved a remarkable titer of 15.8 g/L in batch fermentation using glucose and xylose as carbon sources over 60 h. For salidroside biosynthesis, Bai et al. (2014) successfully synthesized the compound by introducing the glycosyltransferase UGT73B6 from *Rhodiola sachalinensis* into a tyrosol-producing *E. coli* strain. Through shake-flask fermentation with glucose as the substrate, they achieved a salidroside yield of 56.9 mg/L. These studies highlight the feasibility of microbial biosynthesis for producing eriodictyol and salidroside. Future efforts should focus on pathway optimization, metabolic flux regulation, and bioprocess improvements to enhance yields and enable industrial-scale production.

## 4 Conclusion and future prospects

Tyrosine and its aromatic derivatives have various functions and are widely used in the fields of chemical industry, medicine, and food. However, traditional methods, such as chemical synthesis or plant extraction, face problems such as scarce raw material resources, high pollution in the production process, and high energy consumption, making them unsustainable, severely restricting industrial development, and unable to meet market demand. Using renewable biomass as a substrate and producing these high-value-added chemicals through microbial fermentation has energy-saving, environmental protection, and sustainable advantages compared with existing production methods and has considerable commercial prospects.

Although microbial fermentation holds promise for the production of tyrosine-derived products, most of these endeavors remain in the research and development phase. The fact that only a limited number of studies have progressed to the point of potential industrial application indicates that significant challenges persist.

First, tyrosine and its derivatives, which are often metabolites with benzene rings, are inherently toxic to cells. This results in competitive contradiction between cell growth and product synthesis. To address this issue, the author proposed selecting gram-positive host cells with improved tolerance and employing adaptive evolution techniques to optimize the tolerance of chassis cells. For instance, the gram-positive bacterium *C*. *glutamicum* demonstrates superior tolerance to phenylethanol in comparison to the gram-negative *E*. *coli*, thereby establishing itself as a comparatively more suitable candidate for phenylethanol production (Zhu et al., 2023). Through adaptive evolution, an evolved strain *E*. *coli* was obtained, which demonstrated a 10.0% increase in OD_600_ and achieved a tyrosol titer of 3.3 g/L (Zeng et al., 2024).

Second, the metabolic pathways of numerous tyrosine derivatives are derived from eukaryotes, and the activity of eukaryotic enzymes is low if expressed in prokaryotes (*E. coli*), which limits the efficiency of product synthesis. Strategies such as codon optimization of genes, co-expression of molecular chaperones, and utilization of different expression vectors can be employed to improve the expression levels of heterologous enzymes. In addition, employing directed evolution technology for the molecular transformation of enzymes is an effective strategy for enhancing the catalytic activity of heteroenzymes. In particular, the advent of AlphaFold3 has facilitated the prediction of enzyme structures, thus simplifying the process of enzyme modification (Abramson et al., 2024). AlphaFold3 can predict the 3D structure of enzymes involved in metabolic pathways, aiding in the design of more efficient or novel enzymes. This is crucial for engineering microbes to produce high-value chemicals, biofuels, or pharmaceuticals more efficiently. In addition, optimizing entire pathways often requires a detailed understanding of how enzymes interact within the cellular context. AlphaFold3’s accurate protein structure predictions can help identify key interaction sites between enzymes and metabolites, guiding pathway optimization.

Third, the primary factor limiting the large-scale production of tyrosine and its derivatives is low yield. Existing engineered strains primarily focus on genetic modifications targeting local tyrosine branches and derivative pathways. It is essential to manage the equilibrium between biomass formation, metabolite production, and redox systems systematically. Systems biology tools, encompassing transcriptomics, proteomics, metabolomics, genomics, fluxomics, and genome-scale metabolic models (GSMMs), transform intricate biological behaviors into tangible data and information. The application of these tools allows us to analyze cellular metabolism from multiple dimensions, offering richer information for the screening of genetic targets and thereby enhancing the efficiency of developing high-performance chassis cells.

## Abbreviations

AAAs, Aromatic amino acids; EMP: glycolytic pathway; PEP, phosphoenolpyruvate; MOPS, 3-morpholino propanesulfonic acid; L-DOPA, levodopa; MAGE, Multiplex Automated Genome Engineering; PAL, phenylalanine ammonia lyase; PAPS: 3′-phosphoadenosine 5′-phosphosulfate; p-HAP, p-Hydroxyacetophenone; G-6-P, glucose-6-phosphate; DHQ, 3-dehydroquinic acid; DHS, 3-dehydroshikimic acid; E4P, erythrose-4-phosphate; DAHP, 3-deoxy-D-arabinoheptulose-7-phosphate; SA, shikimic acid; S3P, shikimate-3-phosphate; EPSP, 5-enolpyruvoylshikimate 3-phosphate; CHA, chorismic acid; PPH, prephenate; 4-HP, 4-hydroxyphenylpyruvate; TAL, tyrosine ammonia-lyase; GSMMs, genome-scale metabolic models.
